# Performance of Eudragit Coated Whispering Gallery Mode Resonator-Based Immunosensors

**DOI:** 10.3390/s121114604

**Published:** 2012-10-30

**Authors:** Ambra Giannetti, Simone Berneschi, Francesco Baldini, Franco Cosi, Gualtiero Nunzi Conti, Silvia Soria

**Affiliations:** 1 Istituto di Fisica Applicata Nello Carrara (IFAC CNR), Via Madonna del Piano 10, 50019 Sesto Fiorentino, Firenze, Italy; E-Mails: a.giannetti@ifac.cnr.it (A.G.); s.berneschi@ifac.cnr.it (S.B.); f.baldini@ifac.cnr.it (F.B.); f.cosi@ifac.cnr.it (F.C.); g.nunziconti@ifac.cnr.it (G.N.C.); 2 Centro Studi e Ricerche e Museo Storico della Fisica Enrico Fermi, Piazza del Viminale 1, 00184 Roma, Italy

**Keywords:** whispering gallery mode resonators, immunosensors, Eudragit

## Abstract

Whispering gallery mode resonators (WGMR) are an efficient tool for the realization of optical biosensors. A high Q factor preservation is a crucial requirement for good biosensor performances. In this work we present an Eudragit^®^L100 coated microspherical WGMR as an efficient immunosensor. The developed resonator was morphologically characterized using fluorescence microscopy. The functionalization process was tuned to preserve the high Q factor of the resonator. The protein binding assay was optically characterized in terms of specificity in buffer solution.

## 1. Introduction

In optical bioassays, label-based assays exploit the interaction between the analyte under study and a capturing element, labelled with a fluorescent or chemiluminescent molecule. On the other hand, the use of a label very often implies multistep detection protocols (as in the ELISA tests or in sandwich assays) which can complicate the biochemical interaction and can cause sensor cross-sensitivities. On the contrary, in a label-free approach, which is based on the change of the refractive index in the medium surrounding the optical waveguide, does not suffer this inconvenience and offers the possibility to measure the interaction between the capturing element and the analyte directly and in real time, providing the possibility of also investigating dynamic interactions.

Detection of immunoagents and pathogens in a medical context requires the development of both highly sensitive and selective biosensors. This increasing demand for reliable detection of biomolecules has resulted in a large variety of optical label-free biosensors [1]. Among optical biosensors, evanescent wave based sensors are the most outstanding sensor platforms due to their capability of detecting changes induced by the binding of analytes within a submicron penetration depth (10 s to 100 s of nm). Whispering Gallery Mode Resonators (WGMRs) offer a very promising alternative for the development of highly sensitive label-free biosensors [1–6], with the change in *Q* or in resonant wavelength being used for measuring binding phenomena on the WGMR surface. It is apparent that high quality factor *Q* and long recirculation of light in compact WGMRs are the most important features for sensing applications.

The permanence of high *Q* values after the functionalization of the surface is an essential requirement in order to achieve highly sensitive biosensors. There is wide variety of surface chemistry already established for silica based WGMRs [7–9]. Recently, we investigated the effect of polymeric materials on microspherical WGMRs' *Q* factors [10] following a procedure published in [11,12]. In this paper, we have effectively immobilized IgG and studied the resulting functionalized resonator morphologically by means of fluorescence microscopy. Afterwards, we have tested the performance of the WGMR immunosensors in a fluidic cell.

## 2. Experimental Section

### 2.1. Materials

Bovine serum albumin (BSA), ethanol (EtOH), phosphate-buffered saline (40 mM PBS, pH 7.4) and ethanolamine were purchased from Sigma-Aldrich (Milan, Italy). The methacrylic acid/methacrylate copolymer Eudragit^®^L100 (n_D_^20^ = 1.390–1.395) was purchased from Degussa, Röhm Pharma Polymers (Düsseldorf, Germany). The mouse IgG, goat anti-mouse-IgG and alexafluor488-labeled goat anti-mouse-IgG were purchased from Zymed Laboratories, Invitrogen Immunodetection (Milan, Italy). 1-Ethyl-3-[3-dimethylaminopropyl] carbodiimide hydrochloride (EDC) and *N*-hydroxysuccinimide (NHS) were purchased from Pierce (Rockford, IL, USA).

### 2.2. Surface Functionalization and Bioassay Protocol

Microspheres were treated by immersion in 10 mM Eudragit^®^L100 in ethanol for 1 min and then in exposed to air for about 15 min, until complete solvent evaporation had occurred. The polymeric deposition provides carboxylic functional groups (–COOH) on the surfaces, useful for biomolecule immobilization. Once the microspheres were functionalised, they were placed in a flow cell (see Section 2.3) and all the subsequent steps for the preparation of the biological sensing layer were performed: (i) activation of –COOH groups by means of EDC (2 mM) and NHS (5 mM) for 30 min; (ii) immediate covalent immobilization of the antibodies on the microsphere surface, by pumping a solution of 100 mg·L^−1^ antibody (Alexafluor488-labelled goat anti-mouse-IgG when the layer formation was investigated, mouse IgG when the bioassay was performed) in PBS for 1 h; (iii) washing with PBS for 5 min to remove the unreacted antibody. After this step the microspheres with Alexafluor488-labelled goat anti-mouse-IgG were ready for measurements. The microspheres with mouse IgG were subject to further treatments; (iv) inactivation of carboxylic groups with 1 M ethanolamine for 10 min; (v) washing with PBS for 5 min; (vi) surface passivation with 10% BSA in PBS for 15 min in order to reduce/eliminate the non-specific adsorption on the glass surface; (vii) washing with PBS for 5 min; (viii) injection for 30 min of Alexafluor488-labelled goat anti-mouse-IgG 50 mg·L^−1^ in order to monitor optically the specific antigen/antibody interaction.

### 2.3. Experimental Set-up

Microspheres can easily be fabricated directly on the tip of a standard telecom fiber. For this purpose we used a fiber fusion splicer (FITEL S182K). A cleaved tip of the fiber is inserted in one arm of the splicer and a series of arcs are then produced. The tip partially melts and surface tension forces produce the spherical shape. The size of the spheres increases with the number of arc shots, up to a diameter saturation value of around 350 μm [13]. The microspheres used in this work have an average diameter of 260 μm and were stored under vacuum, in order to avoid contamination. The residual fiber stem is then mounted on a translation stage with piezoelectric actuators and a positioning resolution of 20 nm.

**Figure 1.**
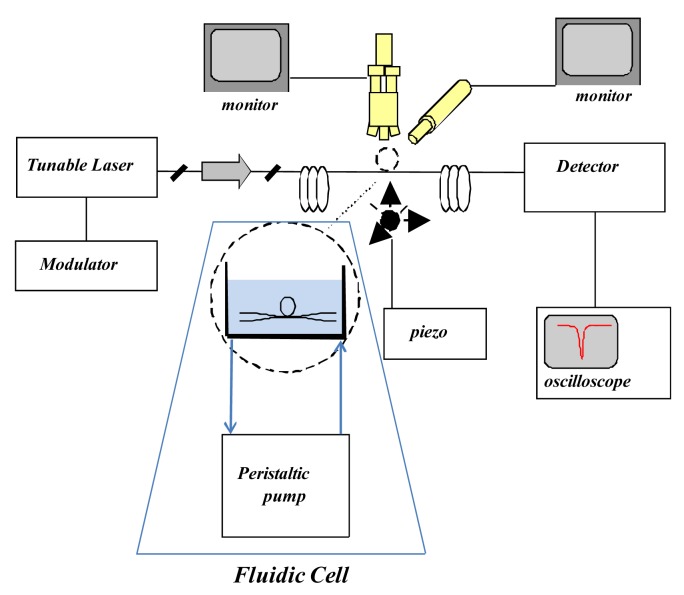
A schematic diagram of the experimental arrangement.

The laser light is coupled to the WGM resonator by means of a tapered fiber of about 3 μm diameter, produced in-house too. We used a microfluidic flow system that incorporates the WGMR and the tapered optical fiber. Our system consists of an open cell of 3 mL of volume with static flow. The tapered fiber was bound to the bottom of the cell by UV adhesive and placed parallel to the flow. The microsphere was then slowly lowered down to the contact point with the thinnest region of the taper and aligned at the centre of the fluidic cell. A peristaltic pump was used for filling the cell and injecting the antigen into the solution. A tunable external cavity laser (NetTest Tunics Plus), characterised by a working wavelength around 1.5 μm and a linewidth of 300 kHz, is finely and slowly (180 Hz) modulated in wavelength around a WGM resonance of a few GHz. The light transmitted through the coupler-WGM resonator system was monitored with the pump off, at the output of the tape, using an amplified In GaAs photodiode detector connected to an oscilloscope, as shown in Figure 1.

The *Q* values were obtained by measuring the resonance linewidth of the WGM modes around 1,550 nm. The *Q* factor of the WGMRs was monitored throughout the functionalization process to ensure that the chemical process and subsequent attachment of proteins preserved its optical performance [9].

## 3. Results and Discussion

### 3.1. Optical Characterization

The different steps of the protocol followed for the immobilization of the capturing element as well as for the bioassay implementation were morphologically characterized using a fluorescence microscope (Nikon Eclipse E600), thanks to the use of labeled antibodies, as described in Section 2.2. Table 1 lists the samples characterized by fluorescence microscopy and their relative *Q* factors measured in buffer solution.

In order to evaluate the distribution of IgG on microspheres, the Alexa488 goat anti-mouse-IgG was immobilized on Eudragit coated microspheres using the protocol described above. The microsphere was immobilized on a microscope slide with a double-sided tape in order to reduce the lateral shift. In order to confirm the bio-recognition event, observations of anti-mouse-IgG modified microspheres incubated with labeled protein solutions were carried out.

**Figure 2.**
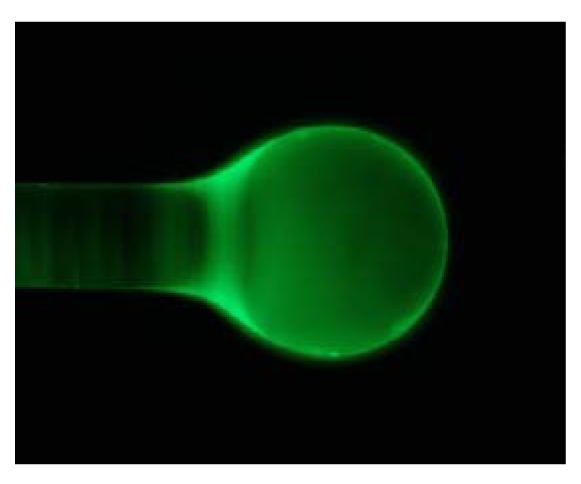
Fluorescent image of the sample anti-mouse-IgG.

**Figure 3.**
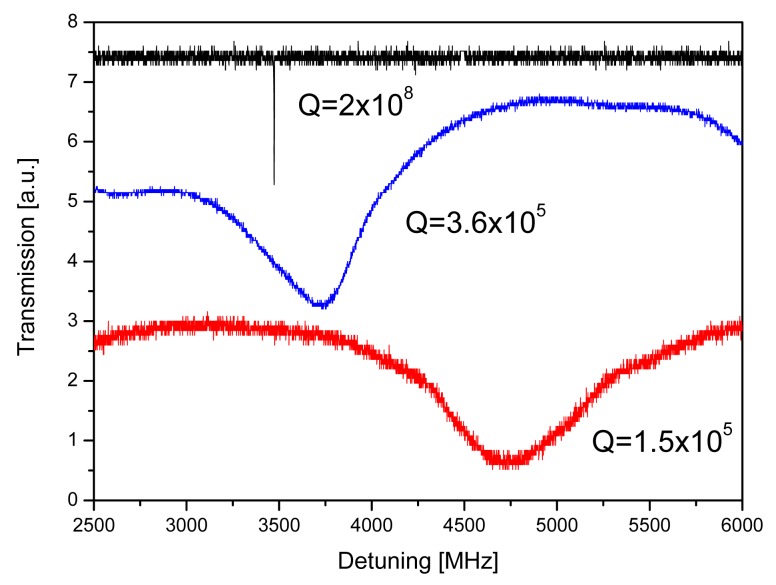
Measured *Q* factor of the NT sample (black line), of a covalent binding of IgG (blue line) and an IgG/anti-IgG interaction (red line).

The anti-mouse-IgG distribution on the microsphere was found to be quite uniform, in particular in the equatorial region, as shown in Figure 2. From optical images and the measured *Q* factor is apparent that Eudragit functionalization induces a uniform modification of the surface of the microspheres. The *Q*-factor preservation allowed efficient protein target recognition, as reported in the optical biosensor detection section [10]. The measured *Q* factor after IgG/anti-IgG binding is 1.5 × 10^5^ at 1,550 nm in PBS. Figure 3 shows the measured *Q* factor for an NT sample, an IgG one and the last one after IgG/anti-IgG binding.

### 3.2. Binding Assay

**Figure 4.**
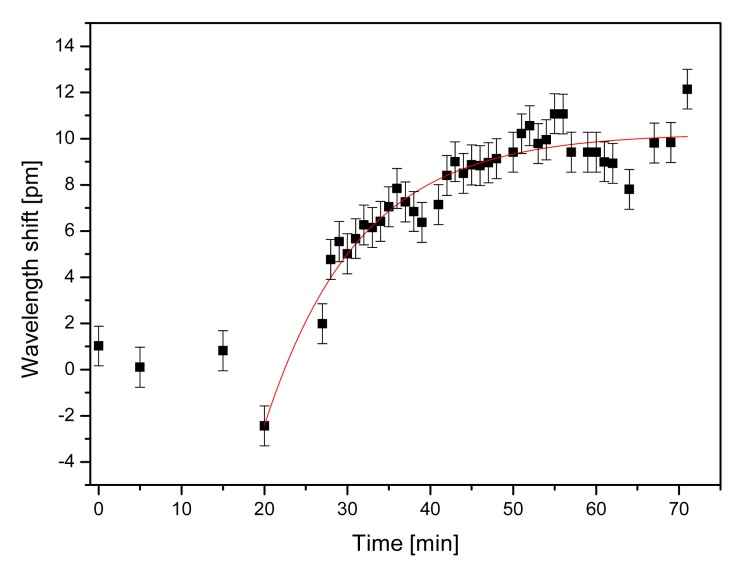
Sensorgram of anti-IgG binding to a WGMR immobilized with IgG in PBS, following the addition of anti-IgG.

In order to study the feasibility of using the Eudragit coated microsphere for immunosensing applications, we carried out a preliminary experiment in which we injected 10 μL of 50 mg·L^−1^ Alexafluor488-labelled goat anti-mouse-IgG so as to monitor the specific antigen/antibody interaction. Figure 4 shows the sensorgram, illustrating that the response of the WGMR immunosensor after goat anti-mouse-IgG injection is specific.

The wavelength shift is first negative due to thermal effects [13,14], but rapidly reverses as the resonant wavelength begins to increase and saturates with time. The sensor to sensor performance was highly reproducible when using freshly prepared samples. The experiments were repeated several times with similar results. For the IgG/anti-IgG binding experiment, we have measured a wavelength shift of about 10.2 ± 0.3 pm. Wavelength shifts are related to surface density of bound molecules by the following equation [15,16]:

(1) $$\delta \lambda /\lambda =\alpha_{\text{ex}}\sigma /[{ɛ}_{0}({{\text{n}}_{\text{s}}}^{2}-{{\text{n}}^{2}}_{\text{m}})\text{a}]$$

where n_s_ and n_m_ are the refractive indices of the microsphere and the medium, respectively, a is the radius of the microsphere, *α_ex_* is the excess of polarizability, and *σ* is the surface density of molecules forming a layer.

The excess of polarizability for IgG can be calculated from [16]:

(2) $$\alpha_{\text{ex}}=2\times {ɛ}_{0}\times {\text{n}}_{\text{m}}\times (\text{dn}/\text{dc})\times {\text{M}}_{\text{w}}/{\text{n}}_{\text{A}}$$

where dn/dc is the differential refractive index of a protein solution in water, ∼0.184 cm^3^/g [17], M_w_ = 150 kDa is the molecular weight of IgG and n_A_ is the Avogadro's number. With α_ex_ = 4πε _0_ × 9.85 × 10^−21^ cm^3^, a = 130 μm (the average microsphere radius used in these experiments) and δλ = 10.2 pm, the anti-IgG surface density is estimated to be 2.25 × 10^11^ cm^−2^, which is close to a compact layer. Although IgG is a globular protein, it is difficult to predict the exact conformation and hence its size when adsorbed to a surface, although we assume that it binds with its largest planar projection in order to reduce the surface energy. Crystallographic and TEM data give an upper limit of approximately 16 to 19 nm [18,19]. Assuming IgG as a roughly spherical molecule of about 17.5 nm of diameter [20], its projected area is then about 240 nm^2^. The maximum fractional area coverage is estimated to be 0.55 for random packing of spheres [21] which means that σ_max_ = 2 × 10^11^ cm^−2^ for IgG. Thus, our IgG layer is about the theoretical level.

Binding of kinetics could be fit to a simple model. The signal of the WGMR biosensor, neglecting the dissociation rate constant, can be modelled by a differential equation, whose solution is a simple exponential [22]:

(3) $$\delta \lambda =\delta \lambda_{\max}[1-\exp(-k_{\text{a}}\text{Ct})]$$

where C is the concentration and *k_a_* is the association constant. In this simple model, we have neglected transport phenomena. From the experimental data, assuming that binding is saturated with the used concentration, we obtained that the association constant *k_a_* is about 1.9 × 10^5^ M^−1^s^−1^ for IgG, which are in good accordance with published results for IgG/anti-IgG binding, based on fiber optics biosensors [23] but 10-fold lower than those obtained using conventional methods [24].

## 4. Conclusions

In conclusion, we have demonstrated a WGMR based immunosensor. We have studied and developed the most suitable chemical protocol in order to obtain a homogeneous thin polymeric layer. Subsequent chemical activation and covalent binding of globular proteins like IgG preserve a value of *Q* high enough for immunosensing applications. We have performed a series of morphological studies in order to assess the quality of the surfaces and determine whether the covalent binding is achieved. It was also shown that WGMR sensors can be used for determining binding kinetics.

## Figures and Tables

**Table 1.** Description of the samples used for optical characterization and their *Q* factor.

**Sample**	**Description**	**Q Factor**
NT	Non-treated sample in air	2 × 10^8^
EU	Eudragit coated sample	5.6 × 10^6^
IgG	Eudragit and Alexa488 mouse IgG sample	3.6 × 10^5^
AntiIgG	Eudragit, mouse IgG and Alexa488 goat anti-mouse IgG sample	1.5 × 10^5^
